# Mental health systems research is urgently needed

**DOI:** 10.1186/1752-4458-1-2

**Published:** 2007-08-09

**Authors:** Benedetto Saraceno

**Affiliations:** 1 Department of Mental Health and Substance Abuse, World Health Organization, 20, avenue Appia 1211 Geneva, Switzerland

## Abstract

Recent developments, including experience related to the development of WHO's World Health Report 2001, the WHO Atlas and the DCP Project related to Mental, Neurological, Developmental and Substance Abuse Disorders, indicate why advancing the interests of mental health is now so compelling.

In order to deliver a high standard of mental health treatment and care WHO emphasizes the adoption of an integrated system of service delivery to address comprehensively the psychosocial needs of people with mental disorders.

Even though the burden is large and increasing, the capacity to reach those in need is poor. This gap cannot be filled just by seeking more funding for mental health, more human resources, or more training. Of course, these aspects are key ingredients but what is often neglected is the need to conceive service delivery rationally.

Mental health professionals' attention should be channeled towards mental health systems and service organization which obviously has consequences in their training which should include more public health knowledge.

We need to know how to plan and organize services and improve the use of scarce financial and human resources in order to reach out to the mental health needs of the general population and to provide effective and humane services to those who need care.

## Editorial

At the present time, the focus on noncommunicable diseases and mental health would appear to be the next natural step in public health priorities. In the case of mental health this is due to the inherent potential of mental health disorders to proliferate as a result of complex and multiple biological, psychological and social determinants.

Recent developments, including experience related to the development of the World Health Organization's (WHO) World Health Report 2001 [1], the WHO Atlas [2] and the Disease Control Priorities Project related to Mental, Neurological, Developmental and Substance Abuse Disorders [3], indicate why the case for advancing the interests of mental health has now become so compelling. Mental health problems already account for more than one-eighth of the global burden of disease and this is likely to increase in the future. The proportion of the global burden of disease attributable to mental, neurological and substance use disorders are expected to rise from 12.3% in 2000 to 16.4% by 2020. Alcohol consumption alone is responsible for 4% of the global burden. More than 150 million persons suffer from depression at any point in time and nearly one million per year commit suicide.

These problems pose a greater burden on vulnerable groups such as people living in absolute and relative poverty, those coping with chronic diseases and those exposed to emergencies. WHO data from Project Atlas have clearly demonstrated that low and middle income countries at present allocate a very small proportion of their financial and human resources to these areas. The median percentage of governments' health budget earmarked for mental health is as little as 1% in low income countries. One-fifth of all countries and nearly half of all low income countries spend less than 1% of their health budget on mental health. Fifty-four percent of all low and middle income countries have less than one psychiatrist and 52% less than one psychiatric nurse per 100,000 population.

The gap in resources is not confined to their quantity. The quality of resources, the way they are allocated and the service provided are often extremely poor, not only in low and middle income but also in high income countries. For example, in spite of years of debate and solid evidence of the need to reduce substantially the hegemony of psychiatric hospitals and to increase community-based mental health care, nearly two-thirds of all mental health beds still remain in large mental hospitals.

The training of health care professionals in mental, neurological, developmental and substance abuse areas is rudimentary and often confined to drug treatment, leaving the psychosocial needs completely unmet. Health care providers often perpetuate stigma rather than ameliorating it. The mental health infrastructure and services in most countries are grossly insufficient for the huge and growing needs.

In order to deliver a high standard of mental health treatment and care WHO emphasizes the adoption of an integrated system of service delivery which attempts to address comprehensively the full range of psychosocial needs of people with mental disorders. A number of policy recommendations for service organization have been highlighted in the World Health Report 2001. They include 1) shifting care away from large psychiatric hospitals, 2) developing community mental health services and 3)integrating mental health care into general health services. WHO has developed a framework which conceptualizes an optimal mix of services for mental health. It reinforces the idea that no single service will meet all needs, and that what is needed is an optimal mix of a range of services [4]. According to this framework and starting at the top of the pyramid: the least numerous services ought to be mental hospitals and specialist services; the second layer includes formal community mental health services and general hospital-based services; the third layer represents mental health services provided through primary health care; and the fourth layer represents informal community mental health services such as traditional healers, school teachers, village elders and so on.

It is evident from what is stated so far, that in spite of the fact that the burden is large and probably increasing in the future, the capacity to reach all those in need is extremely poor. This gap cannot be filled just by seeking more funding for mental health, or more human resources, or more training. Of course, all these aspects are key ingredients but what is too often neglected is the need to conceive service delivery in a rational way. When the mental health treatment gap is addressed, the emphasis is still on treatment per se as if this were a magic bullet in a vacuum instead of something occurring within the framework of a service organization as a part of a health system guided by rational policies.

The attention of mental health professionals should be channelled much more towards mental health systems and service organization which obviously has consequences in their training which should include more public health knowledge.

The number of scientific journals and consequently scientific papers devoted to treatment is more abundant than the literature devoted to documenting, analysing and assessing mental health services and mental health system development. The plethora of information on treatment and the prevailing clinical perspective should be gently replaced or, at least, balanced by an effort to bring a public health perspective in mental health. In this sense, it is important to have a journal focusing on mental health system development, which has the capacity of networking good practice in service organization, giving voice to successful experiences including those from low and middle income countries, promoting health services research and mental health services assessment.

From the WHO perspective, there is no doubt that this is what is needed today. What is needed is to go beyond the simple statements that there are not enough resources for mental health and that mental health is not sufficiently in the global public health agenda. We all know this but we need to understand why, and to identify the barriers to scaling up effective mental health services globally. To do so we need more research, more knowledge and more evidence.

It is very important to have good, randomized, clinical trials providing evidence about the efficacy of new treatments but it is equally important to have research providing evidence that a mental health system in a given country, region or district is working better than another. In other words, what we urgently need to know is how to plan and organize services and improve the use of scarce financial and human resources in order to reach out to the mental health needs of the general population and to provide effective and humane services to those who need care. The WHO concern is for the poor and vulnerable knowing, however, that the poor and vulnerable are not only in low and middle income countries but also in several areas of middle and high income countries.

I personally salute the International Journal of Mental Health Systems as an instrument that will facilitate the future endeavours of those who believe that mental health should be higher on the public health agenda and on the global development agenda and WHO firmly believes this.

## References

[B1] The World Health Report, 2001 (2001). Mental Health: New understanding, new hope. World Health Organization.

[B2] Mental Health Atlas, 2005 (2005). World Health Organization.

[B3] (2006). Disease control priorities related to mental, neurological, developmental and substance abuse disorders. World Health Organization.

[B4] Funk M, Saraceno B, Drew N, Lund C, Grigg M (2004). Mental health policy and plans: Promoting an optimal mix of services in developing countries. International Journal of Mental Health.

